# Evolution of the Toxins Muscarine and Psilocybin in a Family of Mushroom-Forming Fungi

**DOI:** 10.1371/journal.pone.0064646

**Published:** 2013-05-23

**Authors:** Pawel Kosentka, Sarah L. Sprague, Martin Ryberg, Jochen Gartz, Amanda L. May, Shawn R. Campagna, P. Brandon Matheny

**Affiliations:** 1 Department of Biochemistry and Cellular and Molecular Biology, University of Tennessee, Knoxville, Tennessee, United States of America; 2 Department of Psychology, University of Tennessee, Knoxville, Tennessee, United States of America; 3 Department of Ecology and Evolutionary Biology, University of Tennessee, Knoxville, Tennessee, United States of America; 4 MITZ Merseburg, Merseburg, Germany; 5 Department of Chemistry, University of Tennessee, Knoxville, Tennessee, United States of America; University of California Riverside, United States of America

## Abstract

Mushroom-forming fungi produce a wide array of toxic alkaloids. However, evolutionary analyses aimed at exploring the evolution of muscarine, a toxin that stimulates the parasympathetic nervous system, and psilocybin, a hallucinogen, have never been performed. The known taxonomic distribution of muscarine within the Inocybaceae is limited, based only on assays of species from temperate regions of the northern hemisphere. Here, we present a review of muscarine and psilocybin assays performed on species of Inocybaceae during the last fifty years. To supplement these results, we used liquid chromatography–tandem mass spectrometry (LC–MS/MS) to determine whether muscarine was present in 30 new samples of Inocybaceae, the majority of which have not been previously assayed or that originated from either the tropics or temperate regions of the southern hemisphere. Our main objective is to test the hypothesis that the presence of muscarine is a shared ancestral feature of the Inocybaceae. In addition, we also test whether species of Inocyabceae that produce psilocybin are monophyletic. Our findings suggest otherwise. Muscarine has evolved independently on several occasions, together with several losses. We also detect at least two independent transitions of muscarine-free lineages to psilocybin-producing states. Although not ancestral for the family as a whole, muscarine is a shared derived trait for an inclusive clade containing three of the seven major lineages of Inocybaceae (the Inocybe, Nothocybe, and Pseudosperma clades), the common ancestor of which may have evolved ca. 60 million years ago. Thus, muscarine represents a conserved trait followed by several recent losses. Transitions to psilocybin from muscarine-producing ancestors occurred more recently between 10–20 million years ago after muscarine loss in two separate lineages. Statistical analyses firmly reject a single origin of muscarine-producing taxa.

## Introduction

The alkaloid muscarine, an ammonium quaternary compound that stimulates the parasympathetic nervous system of animals, is found in clinically significant amounts in basidiomata (fruitbodies) of several distantly related groups of mushroom-forming fungi (*Clitocybe* sensu lato, *Mycena*, *Omphalotus*, *Inocybe*) [1], [2]. Muscarine binds to acetylcholine receptors and induces a characteristic suite of symptoms that include profuse sweating, lacrimation, and bradycardia. These symptoms are generally expressed quickly within two hours after consumption. The toxin is particularly widespread in basidiomata of species of *Inocybe* sampled from North America and Europe [3]–[13].

Species of *Inocybe* may be mistaken for, or mixed with, other desirable species for consumption, perhaps in part because mushrooms that contain muscarine tend to fruit in urban environments and therefore are more easily collected for the table or grazed by young children [2]. A flush of *I. patouillardii* (now known as *I. erubescens*) in Germany in June 1963 led to mass poisonings in humans [14]. Dogs are particularly susceptible to muscarine poisoning with some cases resulting in death [15], [16]. Over a thirty-year period in the United States, four reported cases of human poisonings and ten in dogs were confirmed due to muscarine intoxication [17]. However, most mushroom intoxications in the United States fail to be ascribed to any particular fungal species. Outside the United States, muscarine intoxication cases continue to be documented, including a recent human fatality [18]–[20]. Fortunately, poisonings are rarely lethal in humans, and atropine may be administered to patients to block muscarine stimulation [2], [21], [22].

Psilocybin, a tryptamine alkaloid that acts on serotonin 5-_HT2A/C_ receptor sites and induces hallucinations [23], was first demonstrated in *Inocybe* in the 1980s [3], [24], [25]. Several related psychotropic toxins–norbaeocystin, baeocystin and psilocin–tend to co-occur with psilocybin. A fifth, aeruginascin, an indole alkaloid and trimethylammonium analogue of psilocybin, was recently discovered in the European species *I. aeruginascens* [26]. This species has also been shown to lack muscarine [27]. Overall, six species of *Inocybe*, five of which correspond to section *Lactiferae*, produce psilocybin and have been shown to lack muscarine (*I. coelestium*, *I. corydalina*, *I. erinaceomorpha*, *I. haemacta*, and *I. tricolor*). However, the classification of *I. aeruginascens* within subgenus *Inocybe* is not known. Species of section *Cervicolores* (viz, *I. calamistrata* and allies) lack muscarine as well but do not produce psilocybin or other hallucinogenic compounds [3].

Although muscarine has been known as a toxin in *Inocybe* for nearly 90 years, methods used for its detection have varied widely. In 1920 muscarine was reported as the main toxin in *I. rimosa* using physiological tests on the heart of a frog [28]. Later, muscarine was isolated as a tetrachloraurate salt from *I. patouillardii* [29], and then again later from *I. fastigata* and *I. umbrina* [30]. A paper chromatographic method was used in the early 1960s [5] to determine muscarine concentrations in various species of *Inocybe*. About the same time rat lacrimation bioassays were also explored [9]. Later, chemotaxonomic tests were employed to assay the presence of muscarine in selected species of *Inocybe* [11]. During the 1980s and 1990s significant advances were made that discovered stereoisomers of muscarine and the presence of psilocybin and other psychotropic compounds in species of *Inocybe* that lacked muscarine [3], [4], [7], [12], [13], [24], [25], [27], [31]. These differences in detection methods produced some conflicting information about presence/absence of muscarine in various species of Inocybaceae.

Few studies, mostly of the genus *Inocybe*, have examined the distribution of muscarine within a systematic framework to determine the evolutionary significance of the toxin [5], [6]. Previously, it was hypothesized that the presence of muscarine had little taxonomic correlation [5]. However, these authors suggested the presence of muscarine may not be entirely random taxonomically [13]. Unfortunately, these studies were hindered by lack of robust phylogenetic hypotheses against which to map the distribution of muscarine-containing species. At present, the evolution of the toxin in the Inocybaceae is ambiguous, but if ancestral would serve as a synapomorphy for the family, which would further warrant separation from the Crepidotaceae [32], the sister group of the Inocybaceae.

Kuyper [33] suggested that muscarine had multiple origins and/or multiple losses within the genus *Inocybe*, but this hypothesis has never been explicitly tested. Thus, it is unclear during the course of evolution of the family where, and when, any origins or losses occurred. Unfortunately, no species from the tropics and southern hemisphere, including species of *Auritella* and *Tubariomyces*, two recently described genera in the family, have been assayed for the presence of muscarine and psilocybin.

Here we present a literature review of species of Inocybaceae reported to contain muscarine or psilocybin (the first since 1990 [34]), conduct LC–MS/MS analysis for muscarine presence in 30 species of Inocybaceae sampled primarily outside Europe, and test the hypothesis that possession of muscarine is an ancestral trait of the Inocybaceae. We also examine the number of times psilocybin evolved in the family. To accomplish these objectives, we assembled a dataset of nuclear large subunit ribosomal RNA (LSU) sequences for ca. 500 species of *Inocybe*, *Auritella*, and *Tubariomyces*, the most taxonomically inclusive dataset for the family to date (ca. 500–700 species have been estimated world-wide). We then reconstructed a chronogram for the family to examine patterns of muscarine and psilocybin evolution within a geological timeframe and test hypotheses for single origins of muscarine and psilocybin within a statistical framework.

## Materials and Methods

### Muscarine- and Psilocybin-containing Taxon Datasets

A literature review of muscarine and psilocybin reports in the Inocybaceae was performed (Table S1). To this we added muscarine presence-absence data for 30 new collections (Table 1) including representatives from previously unsampled regions of Asia, Australia, New Zealand, Africa, and the neotropics. Data matrices for presence/absence of muscarine and psilocybin were then compiled for taxa present in our phylogenetic data set (Table S2) in Mesquite 2.73 [35].

**Table 1:** Taxa sampled for LC-MS/MS muscarine assays, specimen-voucher information, geographic origin, weights of samples, and nuclear large subunit ribosomal RNA (LSU) sequences in GenBank.

Taxa	Field collection numberand/or herbariumvoucher	Geographicorigin	Extract 1(g)	Extract 2(g)	LSU GenBankaccession no.
*Auritella brunnescens* Matheny & Bougher,nom. prov.	PBM3173 (TENN065742)	Australia: New South Wales	0.011	0.012	JQ313558
*Auritella serpentinocystis* Matheny,Trappe & Bougher	PBM3188 (TENN063641)	Australia: New South Wales	0.019	0.014	JQ313559
*Inocybe* aff. *Fibrillosibrunnea*O.K. Mill. & R.N. Hilton	PBM3245 (TENN065741)	Australia: Tasmania	0.019	0.017	HQ832456
*I.* aff. *fraudans* (Britzelm.) Sacc.	MTS2276 (UC)	USA: California	0.021	0.020	EU433887 (JFA11831)
*I. appendiculata* Kühner	SAT-00-261-55 (WTU)	USA: Washington	0.022	0.022	JN974946
*I. caerulata* Matheny, G. Gates & Bougher,nom. prov.	PBM3127 (TENN063699)	New Zealand	0.026	0.017	JQ313560
*I.* cf. *graveolens* (E. Horak) Garrido	PBM3398 (TENN065746)	Australia: Tasmania	0.021	0.018	JQ313561
*I. chondroderma* Stuntz ex Matheny,Giles & Norvell	PBM2027 (WTU)	USA: Washington	0.020	0018	JN974967 (PBM1776)
*I. grammata* Quél. & Le Bret.	PBM2558 (TENN062401)	USA: New Hampshire	0.031	0.023	JQ313562
*I. granulosipes* Cleland	PBM3240 (TENN065744)	Australia: Tasmania	0.029	0.016	JQ313563
*I. granulosipes* Cleland	PBM3363 (TENN065747)	Australia: Tasmania	0.017	0.016	−
*I. lanatodisca* Kauffman	PBM2444 (TENN062505)	USA: Massachusetts	0.027	0.037	JQ313564
*I. leiocephala* D.E. Stuntz	PBM1569 (WTU)	USA: Wyoming	0.018	0.032	GQ906703
*I. luteifolia* A.H. Sm.	PBM2642 (TENN062473)	USA: Tennessee	0.025	0.018	EU307814
*I. marginata* Matheny, Aime & T.W. Henkel	MCA3190 (BRG)	Guyana	0.010	0.017	JN642239
*I. misakaensis* Matheny & Watling	BB3453 (PC0088768)	Zambia	0.029	0.021	JQ313565
*I. niveivelata* Stuntz ex Kropp,Matheny & Hutchinson	PBM2337 (WTU)	USA: Washington	0.021	0.020	JQ313566
*I.* aff. *perlata* (Cooke) Sacc.	JV4336 (WTU)	Finland	0.024	0.021	JQ313567
*I. pileosulcata* E. Horak, Matheny& Desjardin, nom. prov.	DED8058 (SFSU, ZT13025)	Thailand	0.019	0.011	EU600838
*I. rimosoides* Peck	PBM2459 (TENN062320)	USA: New York	0.020	0.025	AY702014
*I. scissa* (E. Horak) Garrido	PBM3394 (TENN065745)	Australia: Tasmania	0.025	0.030	JQ313568
*I. spuria* Jacobsson & E. Larss.	BK18089723 (UTC)	USA: Utah	0.021	0.033	EU600868
*I. subexilis* Peck	PBM2620 (TENN062456)	USA: Tennessee	0.011	N/A	EU307845
*I. subochracea* (Peck) Peck	SH083007 (TENN065743)	USA: New Jersey	0.031	0.030	JN974972
*I. tahquamenonensis* D.E. Stuntz	PBM2680 (TENN062505)	USA: New York	0.027	0.022	AY380399(PBM1142)
*I. unicolor* Peck	PBM2974 (TENN062732)	USA: Tennessee	0.023	0.022	JQ313569
*I. vinaceobrunnea* Matheny, nom. prov.( = *I. jurana* (Pat.) Sacc. sensu Hesler	PBM2951 (TENN062709)	USA: Tennessee	0.030	0.036	HQ201353
*I. viscata* (E. Horak) Garrido	PBM3445 (TENN065734)	Australia: Tasmania	0.024	0.032	JQ313570
*I. xerophytica* Pegler	DJL-GUA-159 (TENN065749)	France: Guadeloupe	0.033	0.029	EU600880 (GUA-242)
*Tubariomyces inexpectatus*(M. Villarreal, Esteve-Rav.,Heykoop & E. Horak) Esteve-Rav. & Matheny	AH25500	Spain	0.010	N/A	GU907091

Taxa from the phylogenetic data set were scored for muscarine-psilocybin presence/absence (1/0 data) according to geographic proximity of assayed species, where sequence data were lacking from assayed collections. We employed this strategy to take into account taxonomic uncertainties, whereby taxa were scored as ambiguous. For example, Stijve et al. [13] reported presence of muscarine in a European sample of *I. flocculosa*; our molecular data set contains a sequence of *I. flocculosa* from Norway, so this tip was scored as positive (1) for muscarine. However, Gurevich et al. [7] report the presence of muscarine in Eurasian *I. umbratica*. We scored the presence of muscarine as positive (1) in our European sample of *I. umbratica* but scored our North American sample as ambiguous because we lack muscarine data for North American materials of this species or species group. In another example, both Malone et al. [9] and Robbers et al. [11] report the presence of muscarine in *I. cinnamomea*, a species endemic to the Pacific Northwest. Our phylogenetic dataset contains a sequence from material of *I. cinnamonea* collected in the Pacific Northwest, the sequence of which is identical to that of the holotype from northern California. Thus, this tip was scored as (1). In yet another example, Western North American and European collections of *I. calamistrata* are reported to lack muscarine [3], [11], [13]. Thus, tips produced from collections sequenced from these geographic regions were scored as (0); however, phylogenetically and geographically distinct lineages of the *I. calamistrata* group (e.g., *I*. aff. *calamistrata* from Costa Rica; *I. mutata* from eastern North America; *I. apiosmota* from eastern North America) were scored as ambiguous for the presence of both toxins. Because the presence of both muscarine and psilocybin in species of Inocybaceae is mutually exclusive [33], taxa confirmed as muscarine-positive were coded psilocybin-negative, and psilocybin-positive taxa were coded as muscarine-negative. To resolve any discrepancies in the literature, we used a simple-majority approach or relied on additional reports. For example, Gartz [36] suggests the presence of psilocybin in *I. calamistrata*. However, both Besl and Mack [3] and Stijve et al. [13] report its absence; thus, European tips of *I. calamistrata* were scored as (0) for psilocybin based on simple-majority rule. Futhermore, it has been suggested by Stijve & Kuyper [37] that the technique used by Gartz [36] is prone to false-positives, hence, further justification for coding European *I. calamistrata* as psilocybin-negative.

### Muscarine Chemical Analysis

Samples of basidiomata from 30 samples of Inocybaceae were flash frozen in liquid nitrogen and then finely ground with a mortar and pestle. The powdered samples, ranging from 10–37 mg, were placed into 1.5 mL microcentrifuge tubes, vortexed for 1 min, and extracted with 300 *µ*L 4∶1 methanol:deionized H_2_O at −78°C for 20 min. The resulting suspensions were centrifuged (13,200 rpm, 16,100 rcf, 5 min) to remove particulates, and a 2 µL portion of the resulting supernatants from each sample was transferred to 200 µL of deionized H_2_O in a separate 300 µL screwcap autosampler vial for analysis by LC–MS/MS as described below. The list of taxa sampled for muscarine detection is provided in Table 1.

A Thermo Electron Surveyor Autosampler Plus was used to inject 10 µL of each sample onto a Phenomenex Gemini reverse phase C_18_ column consisting of fully porous organo-silica with ethane cross-linking, organosilane (5 µm pore size, 110 Å particle size, 150×2 mm column size). High performance liquid chromatography (HPLC) was performed utilizing a quaternary Thermo Electron Surveyor MS Pump Plus with a column temperature of 25°C and a flow rate of 150 µL/min. The eluent was then introduced into a Thermo Electron TSQ Quantum Discovery Max triple quadrupole MS for ion detection. The mobile phases were HPLC grade water (solvent A) and HPLC grade acetonitrile (ACN) (solvent B), and these were used to construct the following 18 min gradient elution profile: *t*) 0 min, 15% solvent A, 85% solvent B; *t*) 2 min, 15% solvent A, 85% solvent B; *t*) 4 min, 95% solvent A, 5% solvent B; *t*) 14, 95% solvent A, 5% solvent B; *t*) 16, 15% solvent A, 85% solvent B; *t*) 18, 15% solvent A, 85% solvent B.

A 0.1 mm internal diameter fused silica capillary was used to introduce samples into the electrospray ionization (ESI) chamber of the triple quadrupole MS. The ESI spray voltage was set at 4500 V for positive ion mode detection. The sheath gas was nitrogen (40 psi), and the inlet capillary temperature was 290°C. The argon collision gas pressure was 1.5 mTorr. Muscarine was detected using selected reaction monitoring (SRM) for the following unique parent mass-to-charge (*m/z*)–product *m/z* combinations at the listed collision energy (in *e*V): 174 *m/z*–57 *m/z*, *e*V 19; 174 *m/z*–115 *m/z*, *e*V 16; 174 *m/z*–60 *m/z*, *e*V 19; 174 *m/z*–97 *m/z*, *e*V 17. The scan time for each SRM was 0.05 s with a scan width of 0.1 *m/z*.

A 2.8 µM standard solution of muscarine was introduced into the MS/MS via direct injection. The four muscarine parent *m/z*–fragments *m/z* pairs that provided the best detection were then determined using the automated MS+MS/MS optimization algorithm provided in Quantum Tune of the Xcalibur v. 2.0.7 software package (Thermo Electron). Once optimized parameters were obtained, the standard sample was introduced onto the column and analyzed via LC−MS/MS using the previously discussed methods. The muscarine standard eluted from the column at a retention time of 1.8 min. Muscarine was scored as present or absent by comparing sample chromatograms to the muscarine standard.

### DNA Extractions, PCR, and Sequencing

We extracted DNA from 24 of the 30 samples used in the muscarine assays. From these we sequenced the LSU region using primers LR0R, LR7, LR3R, LR5, and LR16. DNA extractions, PCR, and sequencing were performed following procedures described in detail by Matheny [32] and Judge [36]. These sequences, or those from conspecific collections, were used for phylogenetic analysis. All new sequences have been submitted to GenBank, a data depository of the National Center for Biotechnology Information (NCBI).

### Phylogenetic Analysis and Ancestral State Reconstructions (ASR)

We assembled an alignment of 497 LSU sequences using MacClade [38] to test the hypothesis that the presence of muscarine is ancestral in the Inocybaceae. This alignment is composed of LSU sequences produced primarily from studies [32], [39]–[45]. BEAST 1.6.1 [46] was used to generate a time calibrated phylogenetic estimate. Five independent runs were executed in BEAST using Markov chain Monte Carlo (MCMC) sampling for 50 million generations, sampling trees and other parameters every 5000 generations. A General-Time-Reversible (GTR) model was used for nucleotide substitution rates in combination with parameters that estimate the proportion of invariable sites and gamma distributed rate heterogeneity to account for substitution rate variation among nucleotide sites. Model selection for molecular evolution was based on Matheny et al. [39] and Ryberg & Matheny [44]. Based on a time calibrated Basidiomycota dataset [47], a normal distributed age prior with a mean of 94 My (million years) and a standard deviation (SD) of 15 My was set for the root height (split between Inocybaceae and the outgrop *Cortinarius*). In addition we applied a normal distributed age prior with a mean value of 50.3 My (SD 12) for the split between Inocybaceae and its sister group, the Crepidotaceae. A Yule model was used as a prior for the distribution of node heights. Tracer 1.5 [46] and AWTY [48] were used to evaluate if convergence had been reached and how many generations to discard as the burn-in.

Parsimony ancestral state reconstruction (ASR) of muscarine and psilocybin production was performed in Mesquite 2.73 [35]. An ASR analysis was performed for each tree in the posterior distribution produced by BEAST. To evaluate whether the distribution of the traits was significantly dependent on the phylogeny, the traits were randomized between the tips (excluding tips for which there were no data), and the parsimony score was calculated in the R package phangorn [49]. One hundred randomizations were performed for each tree in the posterior distribution, and these were compared to the parsimony score for the observed data [50], [51].

### Testing for Monophyly of Muscarine- and Psilocybin-containing Inocybaceae

Two approaches were used to evaluate if muscarine and psilocybin, respectively, are restricted to monophyletic groups. The first approach compared the maximum likelihood of trees constrained to be monophyletic for muscarine and psilocybin, respectively, with the maximum likelihood of the unconstrained tree. Taxa for which the toxin presence was unknown were left unconstrained in all analyses. The maximum likelihood analyses were performed under the GTR plus gamma and invariable sites model, and the tree topologies were compared using the Shimodaira-Hasegawa test (SH-test) [52] in RAxML 7.2.8 [53]. The second approach compared hypotheses of monophyly of muscarine and psilocybin-containing species, respectively, with non-monophyletic alternatives using posterior probabilites. This was done using the APE [54] package in R [54] analyzing the combined post burn-in posterior distributions from the BEAST analyses. Taxa for which muscarine and psilocybin presence were unknown were excluded from each tree in the posterior distribution. The proportion of trees with the group as monophyletic was compared to the proportion of trees where it was not.

## Results

### LC−MS/MS Muscarine Assays

**Figure 1:**
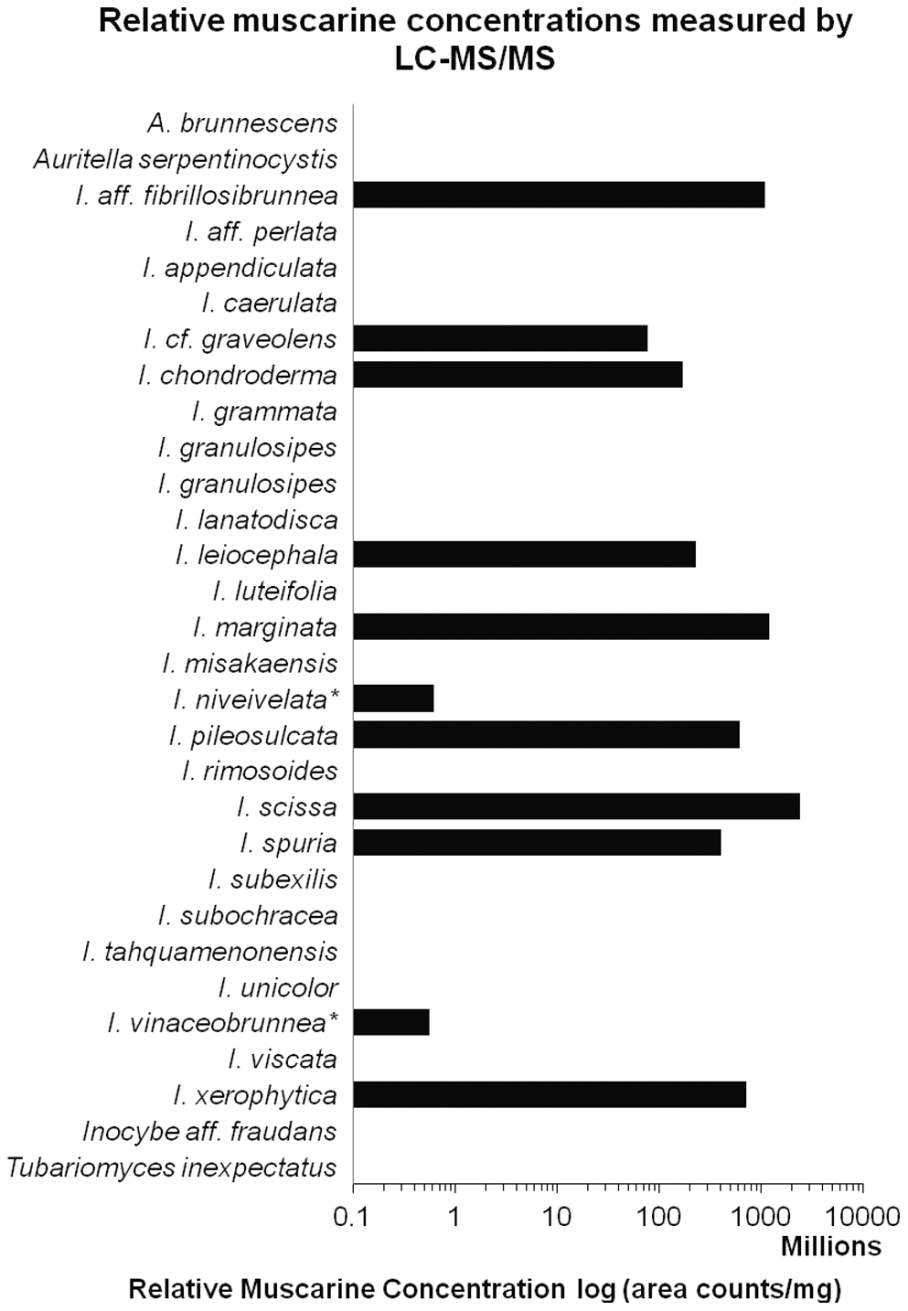
Relative muscarine concentrations measured by liquid chromatography-tandem mass spectrometry. The concentration of muscarine for each sample was determined from the ion counts measured using SRM 174 *m/z*–57 *m/z*. Biological duplicates were used for extraction, and each sample was also injected in duplicate. These four measurements were then averaged. Note: the data reported above have been divided by 10^6^ to minimize the display magnitude of the x-axis units and better highlight the relative amounts between samples. Two species (marked with an *) contained less than 1% of the muscarine concentration of the others. While this amount may not be clinically relevant, the validity of the measurement was confirmed using alternate SRMs.

Of the 30 species we assayed, eleven species of *Inocybe* tested positive for presence of muscarine (Fig. 1). Samples of *I. niveivelata* and *I. vinaceobrunnea* nom. prov. ( = *I. jurana* sensu Hesler), however, are likely clinically insignificant. Muscarine concentrations (normalized) for each sample are shown. Concentrations of muscarine are highest in the Australian-New Zealand species *I. scissa*. The next highest concentrations were detected in *I.* aff. *fibrillosibrunnea* and *I. marginata*, but these samples contained only about half the concentration of muscarine as detected in *I. scissa*. Muscarine detection results from samples of *I. subexilis* (negative), *I. leiocephala* ( = *I. subbrunnea*) (positive), and *I. grammata* ( = *I. albodisca*) (negative) are consistent with earlier findings [5], [6], [11].

### Synopsis of Muscarine- and Psilocybin-containing Species of Inocybaceae

Based on a literature review and the addition of 30 samples here, 99 species of Inocybaceae (96 *Inocybe*, two *Auritella*, one *Tubariomyces*) have been assayed for the presence of muscarine after excluding taxonomic redundancies (Table S1). Of these 99 taxa, 68 (also excluding taxonomic redundancies) have been reported to contain muscarine. Five species from Europe are reported to contain psilocybin, and all lack muscarine.

Considering their phylogenetic placement, six species of subgenus *Mallocybe* (Mallocybe clade) have been assayed. Of these, *I. agardhii*, *I. dulcamara*, *I. caesariata* [55], and *I. malenconii* have been reported as muscarine positive. However, *I. terrigena* and *I. unicolor* lack muscarine. Within section *Rimosae* s. str. (Pseudosperma clade), five species have been assayed. Of these, *I. rimosa*, *I. niveivelata*, *I. sororia*, and *I. spuria* are muscarine positive. Only *I. perlata* is reported to lack muscarine. Twelve species of section *Cervicolores* and the ‘maculata clade’ [43] (two major lineages of the Inosperma clade) have been assayed and included in our phylogenetic analysis. Of these, only three species are muscarine positive: *I. erubescens*, *I. maculata*, and *I. vinaceobrunnea* nom. prov. (the latter likely clinically insignificant). *Inocybe adaequata*, *I. bongardii*, *I. calamistrata*, *I. cervicolor*, *I. cookei*, *I. hirsuta* var. *maxima*, *I. lanatodisca*, *I. misakaensis*, and *I. rimosoides* are nine species within the Inosperma clade that lack muscarine. In subgenus *Inocybe* (Inocybe clade) numerous species are reported as muscarine positive. However, the following lack muscarine and psilocybin: *I. appendiculata*, *I. fraudans*, *I*. aff. *fraudans*, *I. godeyi*, *I. grammata* ( = *I. albodisca*), *I. granulosipes*, *I. incarnata*, *I. luteifolia*, *I. nigrescens*, *I. subexilis*, *I. tahquamenonsis*, *I. viscata*, and *I. xanthomelas*. Species of *Auritella* and *Tubariomyces* sampled here also lack muscarine.

The following species in Table S1 lack muscarine but possess psilocybin: *I. aeruginascens*, *I. coelestium*, *I. corydalina*, *I. haemacta*, and *I. tricolor*. All five psilocybin-containing species appear to be endemic to Europe. One specimen from California reported as *I. bohemica* nom. prov. represents a distinct North American species in the *I. corydalina* group and features the typical blue-green discoloration on the pileus and stipe of basidiomata as in European *I. corydalina*. Given this taxon is nested within what is otherwise a European group of hallucinogenic species, we scored it positive (1) for psilocybin presence. Scoring this taxon as ambiguous for psilocybin made no difference in the number of reconstructed gains of the trait.

We did record several discrepancies of the presence or absence of muscarine in the following species: *I. albodisca*, *I. cervicolor*, *I. curvipes*, *I. dulcamara*, *I. pelargonium*, *I. picrosma*, *I. praetervisa*, *I. serotina*, and *I. xanthomelas* (Table S1). Of these taxa, it seems reasonable that muscarine is indeed lacking from *I. albodisca*, *I. cervicolor*, and *I. xanthomelas* due to a majority or more recent studies that confirm the absence of the toxin. Confusion over species recognition may complicate evaluations of the presence or absence of muscarine in the other taxa listed above. Multiple exemplars of these species should be re-evaluated. One discrepancy was noted for the presence of psilocybin in *I. calamistrata* [36], which has since been demonstrated as psilocybin negative [37].

Previous reports sampled taxa exclusively from Europe or North America. Two species of *Auritella* (*A. brunnescens* nom. prov. and *A. serpentinocystis*), both from Australia, lack muscarine. Of four tropical taxa sampled in this study, three produce muscarine (*I*. *marginata* from Guyana, *I. xerophytica* from the Caribbean, and *I. pileosulcata* nom. prov. from Thailand); the tropical species *I. misakaensis* from Zambia lacks muscarine, but the absence of this toxin is typical for species of section *Cervicolores* to which *I. misakaensis* belongs. Of six temperate Australian and New Zealand species, three (*I.* aff. *fibrillosibrunnea*, *I. scissa*, and *I.* cf. *graveolens*) contain muscarine; three others (*I. caerulata* nom. prov., *I. granulosipes* and *I. viscata*) do not. In addition, this is the first study to assay a species of *Tubariomyces* [42] for presence of muscarine. The type of this genus from mediterranean Spain (*T. inexpectatus*) is confirmed to lack this alkaloid.

### Muscarine is an Older Molecule than Psilocybin but Both are Phylogenetically Conserved

**Figure 2:**
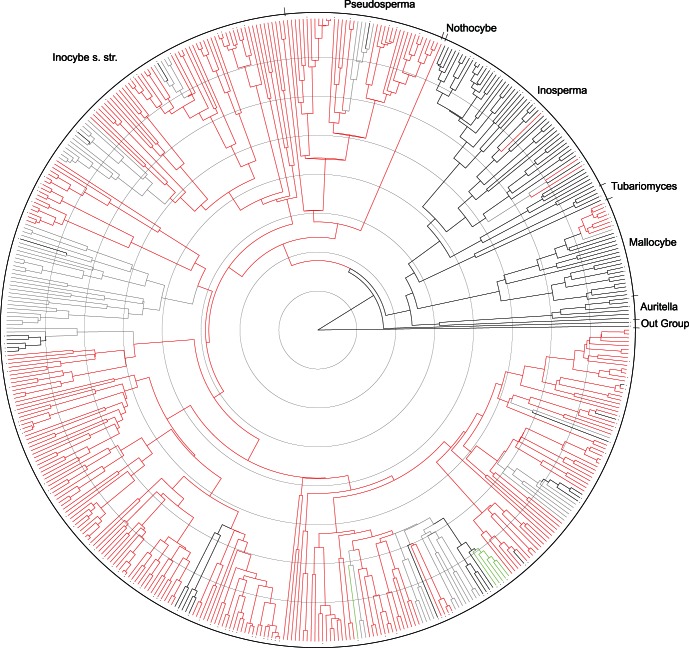
Phylogeny of the Inocybaceae and ancestral state reconstruction of the evolution of muscarine and psilocybin; red indicates the presence of muscarine, green indicates the presence of psilocybin, gray is ambiguous for muscarine, and black indicates the lack of muscarine and lack of psilocybin. Circles indicate time intervals of 10 million years. Dots next to tips indicate species that have been assayed for either muscarine or psilocybin. Major clades of Inocybaceae following [32] are labeled.

The first 30 million generations of each of the five BEAST runs were discarded as burn-in resulting in a final posterior tree distribution of 20 000 trees. The topology of the tree is largely in agreement with previous multigene analyses [32], [44]. The randomization test indicates that both muscarine and psilocybin are significantly dependent on the phylogeny, that is, both are phylogenetically conserved (average P<<0.001). Fig. 2 illustrates the evolution of muscarine- and psilocybin-containing taxa based on an ancestral state reconstruction analysis (Fig. S1 displays a more detailed version of Fig. 2). Muscarine has evolved on multiple occasions but appears to have first originated about 60 million years ago. Psilocybin is present in two independent lineages with each transition occurring much more recently between 10–20 million years ago.

### Polyphyly of Muscarine and Psilocybin-Containing Taxa

The SH-test demonstrates that the most likely tree in which muscarine-containing species are enforced to be monophyletic is significantly worse than the unconstrained tree (*p*<0.01). The most likely tree with psilocybin-containing taxa enforced as monophyletic is not significantly worse than the unconstrained tree (*p*>0.05). Given the conserved nature of the SH-test [52], we also filtered the proportion of trees in which psilocybin-containing (and muscarine-containing) taxa were monophyletic. None of the trees sampled in the posterior distribution were consistent with these taxonomic constraints. Thus, the posterior odds strongly reject the monophyly of groups containing muscarine or psilocybin.

## Discussion

Muscarine and psilocybin are two toxic-inducing secondary metabolites found widely in distantly related lineages of Agaricales. Muscarine induces a suite of symptoms (viz, excessive sweating, lacrimation, and salivation) in response to stimulation of the parasympathetic nervous system. Muscarine is found mainly in species of Inocybaceae but does occur in clinically significant amounts in other genera, including some species of *Clitocybe* sensu lato, and *Mycena* [1], [4]. Mushroom-forming fungi that contain tryptamine derivates (viz, psilocybin) include species of *Psilocybe*, *Panaeolus*, *Conocybe*, *Inocybe*, *Gymnopilus*, *Lycodperdon*, and *Pluteus* [2]. All seven of these genera are restricted to the Agaricales with all of them, except for *Pluteus*, nested in the Agaricoid clade in Matheny et al. (2006) [56]. The evolution of these toxins in mushroom-forming fungi has yet to be evaluated, but we would predict each lineage has acquired either of the two toxins independently given known phylogenetic relationships at this time.

Here, we have provided a comprehensive review of species of Inocybaceae assayed for muscarine and psilocybin over the past 50 years, and to this review we add new data from assays of 30 collections, most of these originating outside Europe and North America. Despite our sampling efforts, only ca. 19% (98 of 507 accepted species) of the family has been assayed for the toxin. This percentage is likely to be lower given that the number of species of Inocybaceae may approach 700 [32] and that novel species continue to be described [42], [57]–[60].

During the course of our review, we came across several obstacles. One, comparing results from each study was challenging because different methods were employed that could be expected to provide varying levels of confidence in the reported relative amounts of muscarine. In the 1960s, for example, a bioassay was used that predicted greater amounts of muscarine than chemical assays [9]. Two, we also observed several taxonomic quandaries: *I. flocculosa*, for example, is reported under three different names in the muscarine literature (*I. flocculosa*, *I. lucifuga*, and *I. gausapata*); all three proved positive for muscarine. In addition, it is now known the *I. rimosa* complex is composed of numerous phylogenetic species [42]. As a consequence, we only coded as positive the most reliably identified sample (from northern Europe) rendering five other potentially unique species in the complex as ambiguous. Three, we also observed that muscarine positive results were supplied in different forms. Gurevich et al. [7], for example, employed a +/− system to record results, whereas Stijve [12] used a % yield. As a conservative measure, we refined all of the data into a +/− system and resolved conflicting results by majority-rule. Lastly, in the literature we uncovered a clear geographic bias for taxa sampled from Europe and North America only.

Little taxonomic correlation has been claimed with respect to the taxonomic distribution of muscarine-containing species of Inocybaceae [5], [6]. However, these prior studies lacked a robust phylogenetic perspective to test the hypothesis. Others, however, have suggested the distribution of muscarine may exhibit some taxonomic correlation [13], a result corroborated here on two grounds: (i) the presence of muscarine is ancestral for a large inclusive clade containing three major lineages–*Inocybe* s. str. ( = subgenus *Inocybe*), the Pseudosperma clade ( = section *Rimosae* s. str.), and the *Nothocybe* lineage (a single stem lineage containing an ambiguously identified species from India); and (ii) a randomization test indicates the taxonomic distribution of muscarine is phylogenetically conserved, more so than expected by chance.

A chronogram of our LSU dataset, which includes approximately 500 species, suggests muscarine initially evolved about 60 million years ago but is not ancestral for the family. This result does not support our null hypothesis. Ancestral state analyses document between 10–13 losses of muscarine across the family, but the presence of muscarine is a highly conserved trait for a large, speciose, and inclusive clade including *Inocybe* s. str., the *Nothocybe* lineage, and the *Pseudosperma* clade. While muscarine is present in the most derived group of the *Mallocybe* clade and in three species of the *Inosperma* clade, it was not detected in deeply branching lineages of the *Mallocybe* clade and is absent from species of *Auritella* and *Tubariomyces* among species sampled thus far.

The chronogram also informs us of a minimum of two independent transitions to a psilocybin-containing state, both of which occurred relatively recently during the Miocene between 10 and 20 million years ago, once in a lineage containing *I. tricolor*, *I. haemacta*, and *I. corydalina* and its allies; and independently in *I. aeruginascens*. All of these species are known only from Europe. We speculate that nLSU-rRNA data alone, combined with the conservative SH-test [52], lack the power to reject the alternative hypothesis of the monophyly of psilocybin-containing taxa. Nevertheless, these results reinforce the observation that the loss of muscarine precedes evolutionary gains of psilocybin. Not all muscarine-lacking taxa, however, produce psilocybin. Indeed, psilocybin-containing taxa are quite rare in the family (app. 1% if one accepts a conservative estimate of ca. 500 species).

Two caveats may temper our results. First, assays for these toxins are hardly taxonomically complete. For example, only 19% of species of Inocybaceae have been assayed for muscarine. However, our phylogenetic results here should serve as a springboard from which to target additional or crucial taxa. Second, our results are dependent on a single gene phylogenetic analysis. Despite this limitation, our LSU dataset is the most taxonomically densely sampled phylogeny of the family to date. We are now in a position to predict that species of *Auritella*, *Tubariomyces*, and deeply branching species of the *Mallocybe* clade and *Inosperma* clade lack muscarine altogether and that unknown species nested in the *I. corydalina* group should possess psilocybin.

Areas of future research should focus on the mechanisms of muscarine gain, loss, and expression, as well as potential selection pressures on its maintenance or loss. Other groups besides the Inocybaceae merit investigation and might be more amenable to laboratory experiments (e.g., *Omphalotus*). It is also unclear if muscarine is expressed in mycelia of these fungi; which organisms, if any, are subject to biochemical deterrence; and the extent to which muscarine acts as a biological defense compound. Invertebrates, such as nematodes and insects, do possess muscarinic acetylcholine receptors [61], [62] and thus may be prone to muscarine intoxication.

Lastly, muscarine and psilocybin are both mutually exclusive compounds in species of Inocybaceae. That is, both toxins have not been found to co-occur in a single species. The biochemical pathways that generate the two toxins are quite different and unrelated [63], [64], [4]. Glutamic acid is a pre-cursor to muscarine, whereas tryptophan is a pre-cursor to psilocybin. However, it would appear the loss of muscarine is a prerequisite to a gain of psilocybin in species of *Inocybe*. This observation is supported by placement of one monophyletic group of psilocybing-containing species (*I. corydalina* and closely related allies) within a grade of taxa (*I. fraudans* and allies) lacking muscarine (Fig. 2). While a second psilocybin-containing lineage of *Inocybe* (*I. aeruginascens*) evolved independently, its sister lineage is not known with confidence. *Inocybe glabripes* is indicated here as a possible sister lineage to *I. aeruginascens*, but the toxic status of the former is unknown. Additional research is required to address biochemical or metabolic reasons for this pattern of mutual exclusivity.

## Supporting Information

Figure S1 **Scalable vector file containing the phylogeny from** **Fig. 2** **including posterior probabilities and tip labeling.** Text in blue indicates taxa used in ASR.
(RAR)

Table S1 Species of Inocybaceae for which muscarine or psilocybin have been assayed according to the literature and new results.
(DOCX)

Table S2 Character state scoring for presence-absence (+/−) of clinical amounts of muscarine and psilocybin, and species and GenBank accession numbers used for phylogenetic analysis.
(DOCX)
