# Supplementary material for: Evolution of the Toxins Muscarine and Psilocybin in a Family of Mushroom-Forming Fungi
Source: PLoS One. 2013 May 23;8(5):e64646. doi: 10.1371/journal.pone.0064646 (PMC3662758; doi:10.1371/journal.pone.0064646)
Supplement: Table S1 — Species of Inocybaceae for which muscarine or psilocybin have been assayed according to the literature and new results. (DOCX) [file pone.0064646.s002.docx]

Table S1. Species of Inocybaceae for which muscarine or psilocybin have been assayed according to the literature and new results presented here.

| **Name in literature** | **Current name** | **Clade** | **Muscarine**  **(+/-)** | **Reference** | **Psilocybin**  **(+/-)** | **Reference** |
| --- | --- | --- | --- | --- | --- | --- |
| *Auritella brunnescens* Matheny & Bougher, nom. prov. |  | Auritella | - | This study |  |  |
| *A. serpentinocystis* Matheny, Trappe & Bougher |  | Auritella | - | This study |  |  |
| *Inocybe acuta* Boud. |  | Inocybe | + | [[1](#_ENREF_1)] |  |  |
| *I. adaequata* (Britzelm). Sacc. |  | Inosperma | - | [[2](#_ENREF_2),[3](#_ENREF_3)] | - | [[2](#_ENREF_2),[3](#_ENREF_3)] |
| *I. aeruginascens* Babos |  | Inocybe | - | [[2-4](#_ENREF_2)] | + | [[2-5](#_ENREF_2)] |
| *I. agardhii* (N. Lund) P. D. Orton |  | Mallocybe | + | [[3](#_ENREF_3)] | - | [[3](#_ENREF_3)] |
| *I. agglutinata* Peck |  | Inocybe | + | [[6](#_ENREF_6)] |  |  |
| *I. albodisca* Peck | *I. grammata* Quél. & Le Bret. | Inocybe | + | [[7](#_ENREF_7)] |  |  |
| *I. albodisca* Peck | *I. grammata* Quél. & Le Bret. | Inocybe | - | [[6](#_ENREF_6),[8](#_ENREF_8)] |  |  |
| *I. appendiculata* Kühner |  | Inocybe | - | [[2](#_ENREF_2)]  This study | - | [[2](#_ENREF_2)] |
| *I. boltonii* R. Heim |  | Inocybe | + | [[6](#_ENREF_6)] |  |  |
| *I. bongardi* (Weinm.) Quél. |  | Inosperma | - | [[2](#_ENREF_2),[3](#_ENREF_3),[9](#_ENREF_9)] | - | [[2](#_ENREF_2),[3](#_ENREF_3),[10](#_ENREF_10)] |
| *I. brevispora* Huijsman | *I. soluta* Velen. | Inocybe | + | [[1](#_ENREF_1)] |  |  |
| *I. brunneorufa* Stangl & J. Veselský | *I. calida* Velen. | Inocybe | + | [[1](#_ENREF_1)] |  |  |
| *I. caerulata* Matheny, Gates & Bougher, nom. prov. |  | Inocybe | - | This study |  |  |
| *I. caesariata* (Fr.) P. Karst. |  | Mallocybe | + | [[9](#_ENREF_9)] |  |  |
| *I. calamistrata* (Fr.) Gillet |  | Inosperma | - | [[2](#_ENREF_2),[4](#_ENREF_4),[6](#_ENREF_6)] | - | [[2](#_ENREF_2),[4](#_ENREF_4)] |
| *I. calamistrata* (Fr.) Gillet |  | Inosperma |  |  | + | [[11](#_ENREF_11)] |
| *I. cervicolor* (Pers.) Quél. |  | Inosperma | + | [[9](#_ENREF_9)] |  |  |
| *I. cervicolor* (Pers.) Quél. |  | Inosperma | - | [[2](#_ENREF_2)] | - | [[2](#_ENREF_2)] |
| *I. chondroderma* Stuntz ex Matheny, Giles & Norvell, nom. prov. |  | Inocybe | + | This study |  |  |
| *I. cinnamomea* A.H. Sm. |  | Inocybe | + | [[6](#_ENREF_6),[7](#_ENREF_7)] |  |  |
| *I. coelestium* Kuyper |  | Inocybe | - | [[2](#_ENREF_2)] | + | [[2](#_ENREF_2),[12](#_ENREF_12)] |
| *I. cookei* Bres. |  | Inosperma | - | [[3](#_ENREF_3)] | - | [[3](#_ENREF_3),[5](#_ENREF_5)] |
| *I. corydalina* Quél. |  | Inocybe | - | [[2-4](#_ENREF_2)] | + | [[2-4](#_ENREF_2),[12](#_ENREF_12)] |
| *I. curvipes* P. Karst. |  | Inocybe | + | [[3](#_ENREF_3)] | - | [[3](#_ENREF_3)] |
| *I. decipientoides* Peck | *I. curvipes* P. Karst. | Inocybe | + | [[7](#_ENREF_7)] |  |  |
| *I. decipientoides* Peck | *I. curvipes* P. Karst. | Inocybe | - | [[6](#_ENREF_6)] |  |  |
| *I. dulcamara* (Alb. & Schwein.) P. Kumm. |  | Mallocybe | + | [[3](#_ENREF_3),[9](#_ENREF_9)] |  |  |
| *I. dulcamara* (Alb. & Schwein.) P. Kumm. |  | Mallocybe | - | [[3](#_ENREF_3)] | - | [[3](#_ENREF_3),[10](#_ENREF_10)] |
| *I. eutheles* (Berk, et Br.) Quél. | *I. sindonia* (Fr.) P. Karst | Inocybe | + | [[3](#_ENREF_3)] | - | [[3](#_ENREF_3)] |
| *I. fastigiata* (Schaeff.) Quél. | *I. rimosa* (Bull.: Fr.) P. Kumm | Pseudosperma | + | [[3](#_ENREF_3),[9](#_ENREF_9),[13](#_ENREF_13)] | - | [[3](#_ENREF_3)] |
| *I. fibrillosibrunnea* O.K. Mill. & R.N Hilton aff. |  | Inocybe | + | This study |  |  |
| *I. fibrosoides* Kühner et Bours. |  | Inocybe | + | [[3](#_ENREF_3)] | - | [[3](#_ENREF_3)] |
| *I. flocculosa* (Berk.) Sacc. |  | Inocybe | + | [[2](#_ENREF_2)] | - | [[2](#_ENREF_2)] |
| *I. fraudans* (Britzlm.) Sacc. aff. |  | Inocybe | - | This study |  |  |
| *I. fuligineo-atra* Huijsman |  | Inocybe | + | [[3](#_ENREF_3)] | - | [[3](#_ENREF_3)] |
| *I. fuscidula* Velen. |  | Inocybe | + | [[3](#_ENREF_3)] | - | [[3](#_ENREF_3)] |
| *I. gausapata* Kühner | *I. flocculosa* (Berk.) Sacc. | Inocybe | + | [[7](#_ENREF_7)] |  | [[7](#_ENREF_7)] |
| *I. geophylla* (Fr.) P. Kumm. |  | Inocybe | + | [[7](#_ENREF_7),[9](#_ENREF_9),[13](#_ENREF_13)] | - | [[3](#_ENREF_3),[10](#_ENREF_10)] |
| *I. geophylla* var. *lilacina* Gillet | *I. lilacina* (Peck) Kauffman | Inocybe | + | [[9](#_ENREF_9),[13](#_ENREF_13)] |  |  |
| *I. geophylla* var. *violacea* (Pat.) Sacc. |  | Inocybe | + | [[1](#_ENREF_1)] |  |  |
| *I. godeyi* Gillet |  | Inocybe | - | [[9](#_ENREF_9)] |  |  |
| *I. grammata* Quél. & Le Bret. |  | Inocybe | - | This study |  |  |
| *I. granulosipes* Cleland |  | Inocybe | - | This study |  |  |
| *I. granulosipes* Cleland |  | Inocybe | - | This study |  |  |
| *I.* *graveolens* (E. Horak) Garrido cf. |  | Inocybe | + | This study |  |  |
| *I. griseolilacina* J.E. Lange |  | Inocybe | + | [[2](#_ENREF_2),[6](#_ENREF_6),[7](#_ENREF_7)] | - | [[2](#_ENREF_2)] |
| *I. haemacta* (Berk. & Cooke) Sacc. |  | Inocybe | - | [[2](#_ENREF_2),[4](#_ENREF_4)] | + | [[2](#_ENREF_2),[4](#_ENREF_4),[12](#_ENREF_12)] |
| *I. hirsuta* var. *maxima* A.H. Sm. |  | Inosperma | - | [[6](#_ENREF_6),[8](#_ENREF_8)] |  |  |
| *I. hirtella* Bres. |  | Inocybe | + | [[9](#_ENREF_9)] |  |  |
| *I. hirtelloides* Stangl & J. Veselský |  | Inocybe | + | [[1](#_ENREF_1)] |  |  |
| *I. incarnata* Bres. | *I. fraudans* (Britzelm.) Sacc. | Inocybe | - | [[2](#_ENREF_2)] | - | [[2](#_ENREF_2)] |
| *I. kauffmanii* A.H. Sm. |  | Inocybe | + | [[6](#_ENREF_6),[7](#_ENREF_7)] |  |  |
| *I. lacera* (Fr.) P. Kumm. |  | Inocybe | + | [[3](#_ENREF_3),[6](#_ENREF_6),[7](#_ENREF_7),[13](#_ENREF_13)] | - | [[3](#_ENREF_3)] |
| *I. lanatodisca* Kauffman |  | Inosperma | - | This study |  |  |
| *I. langei* R. Heim |  | Inocybe | + | [[1](#_ENREF_1)] |  |  |
| *I. lanuginosa* (Bull.: Fr.) P. Kumm. |  | Inocybe | + | [[3](#_ENREF_3),[6](#_ENREF_6)] | - | [[3](#_ENREF_3)] |
| *I. leiocephala* D.E. Stuntz |  | Inocybe | + | This study |  |  |
| *I. lilacina* (Peck) Kauffman |  | Inocybe | + | [[6](#_ENREF_6),[7](#_ENREF_7)] |  |  |
| *I. lucifuga* (Fr.) P. Kumm. | *I. flocculosa* (Berk.) Sacc. | Inocybe | + | [[9](#_ENREF_9)] |  |  |
| *I. luteifolia* A.H. Sm. |  | Inocybe | - | This study |  |  |
| *I. maculata* Boud. |  | Inosperma | + | [[3](#_ENREF_3),[9](#_ENREF_9)] | - | [[3](#_ENREF_3)] |
| *I. malenconii* Heim |  | Mallocybe | + | [[3](#_ENREF_3)] | - | [[3](#_ENREF_3)] |
| *I. marginata* Matheny, Aime & Henkel |  | Inocybe | + | This study |  |  |
| *I. misakaensis* Matheny & Watling |  | Inosperma | - | This Study |  |  |
| *I. mixtilis* (Britzelm.) Sacc. |  | Inocybe | + | [[6-8](#_ENREF_6)] |  |  |
| *I. napipes* J.E. Lange |  | Inocybe | + | [[2](#_ENREF_2),[6-8](#_ENREF_6),[13](#_ENREF_13)] | - | [[2](#_ENREF_2)] |
| *I. nigrescens* G.F. Atk. |  | Inocybe | - | [[6-8](#_ENREF_6)] |  |  |
| *I. nitidiuscula* (Britz.) Sacc. |  | Inocybe | + | [[3](#_ENREF_3)] | - | [[3](#_ENREF_3),[5](#_ENREF_5)] |
| *I. niveivelata* Stuntz ex Kropp, Matheny & Hutchinson, nom. prov. |  | Pseudosperma | + | This study |  |  |
| *I. oblectabilis* (Britzelm.) Sacc. |  | Inocybe | + | [[3](#_ENREF_3),[7](#_ENREF_7)] | - | [[3](#_ENREF_3)] |
| *I. obscura* (Pers.) Gill. | *I. cincinnata* (Fr.) Quél. | Inocybe | + | [[3](#_ENREF_3)] | - | [[3](#_ENREF_3)] |
| *I. obscuroides* P.D. Orton | *I. cincinnata* (Fr.) Quél. | Inocybe | + | [[6](#_ENREF_6),[7](#_ENREF_7)] |  |  |
| *I. olympiana* A.H. Sm. |  | Inocybe | + | [[6](#_ENREF_6),[7](#_ENREF_7)] |  |  |
| *I. ovatocystis* Boursier & Kühner | *I. lanuginosa* (Bull.: Fr.) P. Kumm. sensu amer. auct. | Inocybe | + | [[1](#_ENREF_1)] |  |  |
| *I. pallidipes* Ellis & Everh. |  | Inocybe | + | [[6](#_ENREF_6)] |  |  |
| *I. patouillardii* Bres. | *I. erubescens* A. Blytt | Inosperma | + | [[9](#_ENREF_9)] | - | [[5](#_ENREF_5)] |
| *I. pelargonium* Kühner |  | Inocybe | +/- | [[3](#_ENREF_3)] | - | [[3](#_ENREF_3)] |
| *I. perlata* (Cooke) Sacc. aff. |  | Pseudosperma | - | This study |  |  |
| *I. picrosma* D.E. Stuntz |  | Inocybe | + | [[7](#_ENREF_7)] |  |  |
| *I. picrosma* D.E. Stuntz |  | Inocybe | - | [[6](#_ENREF_6)] |  |  |
| *I. pileosulcata* E. Horak, Matheny & Desjardin, nom. prov. |  | Inocybe | + | This study |  |  |
| *I. praetervisa* Quél. |  | Inocybe | - | [[6](#_ENREF_6),[7](#_ENREF_7)] | - | [[3](#_ENREF_3)] |
| *I. praetervisa* Quél. |  | Inocybe | +/- | [[3](#_ENREF_3)] |  |  |
| *I. pudica* Kühner |  | Inocybe | + | [[2](#_ENREF_2),[6](#_ENREF_6),[7](#_ENREF_7),[9](#_ENREF_9),[13](#_ENREF_13)] | - | [[2](#_ENREF_2)] |
| *I. pusio* P. Karst. |  | Inocybe | + | [[1](#_ENREF_1)] |  |  |
| *I. queletii* Konrad |  | Inocybe | + | [[1](#_ENREF_1)] |  |  |
| *I. rimosoides* Peck |  | Inosperma | - | This study |  |  |
| *I. scissa* (E. Horak) Garrido |  | Inocybe | + | This study |  |  |
| *I. serotina* Peck |  | Inocybe | - | [[3](#_ENREF_3)] | - | [[3](#_ENREF_3),[5](#_ENREF_5)] |
| *I. serotina* Peck |  | Inocybe | + | [[3](#_ENREF_3)] | - | [[3](#_ENREF_3),[5](#_ENREF_5)] |
| *I. soluta* Velen. |  | Inocybe | + | [[3](#_ENREF_3)] | - | [[3](#_ENREF_3)] |
| *I. sororia* Kauffman |  | Pseudosperma | + | [[6](#_ENREF_6),[7](#_ENREF_7)] |  |  |
| *I. spuria* Jacobsson & E. Larss. |  | Pseudosperma | + | This study |  |  |
| *I. subbrunnea* Kühner | *I. catalaunica* Singer | Inocybe | + | [[13](#_ENREF_13)] |  |  |
| *I. subdestricta* Kauffman |  | Inocybe | + | [[7](#_ENREF_7)] |  |  |
| *I. subexilis* Peck |  | Inocybe | - | [[8](#_ENREF_8)] |  |  |
| *I. subexilis* Peck |  | Inocybe | - | This study |  |  |
| *I. subochracea* (Peck) Peck |  | Inocybe | - | This study |  |  |
| *I. tahquamenonensis* D.E. Stuntz |  | Inocybe | - | This study |  |  |
| *I. terrifera* J.G. Kühn |  | Inocybe | + | [[6](#_ENREF_6),[7](#_ENREF_7)] |  |  |
| *I. terrigena* (Fr.) Kuyper |  | Mallocybe | - | [[2](#_ENREF_2)] | - | [[2](#_ENREF_2)] |
| *I. trechispora* (Berk.) P. Karst. | *I. mixtilis* (Britzelm.) Sacc. | Inocybe | + | [[2](#_ENREF_2),[3](#_ENREF_3)] | - | [[2](#_ENREF_2),[3](#_ENREF_3)] |
| *I. tricolor* Kühner |  | Inocybe | - | [[4](#_ENREF_4)] | + | [[4](#_ENREF_4),[12](#_ENREF_12)] |
| *I. umbratica* Quél. |  | Inocybe | + | [[3](#_ENREF_3)] | - | [[3](#_ENREF_3),[10](#_ENREF_10)] |
| *I. umbrina* Bres. | *I. assimilata* Britzelm. | Inocybe | + | [[3](#_ENREF_3),[8](#_ENREF_8)] | - | [[3](#_ENREF_3)] |
| *I. unicolor* Peck |  | Mallocybe | - | This study |  |  |
| *I. vinaceobrunnea* Matheny nom. prov. (=*I. jurana* ss. Hesler) |  | Inosperma | + | This study |  |  |
| *I. virosa* K.B. Vrinda, C.K. Pradeep, A.V. Joseph & T.K. Abraham |  | Inosperma | + | [14] |  |  |
| *I. viscata* (E. Horak) Garrido |  | Inocybe | - | This study |  |  |
| *I. xanthomelas* Boursier & Kühner |  | Inocybe | + | [[7](#_ENREF_7)] |  |  |
| *I. xanthomelas* Boursier & Kühner |  | Inocybe | - | [[3](#_ENREF_3),[6](#_ENREF_6),[8](#_ENREF_8)] | - | [[3](#_ENREF_3)] |
| *I. xerophytica* Pegler |  | Inocybe | + | This study |  |  |
| *Tubariomyces inexpectatus* (M. Villarreal, Esteve-Rav., Heykoop & E. Horak) Esteve-Rav. & Matheny |  | Tubariomyces | - | This study |  |  |

1. Bresinsky A, Besl H (1990) A colour atlas of poisonous fungi: A handbook for pharmacists, doctors, and biologists. . London: Wolfe Publishing Ltd.

2. Stijve T, Klan J, Kuyper TW (1985) Occurrence of psilocybin and baeocystin in the genus *Inocybe* (Fr.) Fr. Persoonia 12: 469-473.

3. Gurevich LS, Nezdoĭminogo EL (1992) Psilocybin and muscarine as possible chemotaxonomic markers for the genus *Inocybe* (Fr.) Fr. Mycologija i Phytopathologija 262: 88-97.

4. Besl H, Mack P (1985) Halluzinogene Rißpilze. Z Mykol 51: 183-184.

5. Becker AM, Gurevich LS, Nezdoĭminogo EL, Onoprienko VV, Kozmin YP (1989) A chromatographical study of some indole metabolites in the genus *Inocybe* II. Mycologija i Phytopathologija 23: 129-134.

6. Robbers JE, Brady L, Tyler Jr V (1964) A chemical and chemotaxonomic evaluation of *Inocybe* species. Lloydia 27: 192-202.

7. Malone M, Robichaud R, Tyler Jr V, Brady L (1962) Relative muscarinic potency of thirty *Inocybe* species. Lloydia 25: 231-237.

8. Brown J, Malone M, Stuntz D, Tyler Jr V (1962) Paper chromatographic determination of muscarine in *Inocybe* species. J Pharm Sci 51: 853-856.

9. Stijve T (1982) Het voorkomen van muscarine en muscimol in verschillende paddestoelen. Coolia 25: 94-100.

10. Becker AM, Gurevich LS, Alekseev SM, Nikonov AL (1988) A chromatographical study of some indole metabolites in the genus *Inocybe* I. Mycologija i Phytopathologija 22: 320-324.

11. Gartz J (1986) Nachweis von Tryptaminderivaten in Pilzen der Gattungen *Gerronema*, *Hygrocybe*, *Psathyrella* and *Inocybe*: Detection of Tryptamine Derivatives in Fungi of the Genera *Gerronema*, *Hygrocybe*, *Psathyrella* and *Inocybe*. Biochemie und Physiologie der Pflanzen 181: 275-278.

12. Gartz J (1986) Untersuchungen zum Vorkommen des Muscarins in *Inocybe aeruginascens* Babos. Zeitschrift für Mykologie 52: 359-361.

13. Catalfomo P, Eugster CH (1970) Muscarine and muscarine isomers in selected *Inocybe* species. Helvetica Chimica Acta 53: 848-851.

14. Vrinda KB, Pradeep CK, Joseph A Vijaya, Abraham TK(1996) A new *Inocybe* (Cortinariceae) from Kerla state, India Mycotaxon *57*: 171-174.
