# Supplementary material for: Evolution of the Toxins Muscarine and Psilocybin in a Family of Mushroom-Forming Fungi
Source: PLoS One. 2013 May 23;8(5):e64646. doi: 10.1371/journal.pone.0064646 (PMC3662758; doi:10.1371/journal.pone.0064646)
Supplement: Table S2 — Character state scoring for presence-absence (+/−) of clinical amounts of muscarine and psilocybin, and species and GenBank accession numbers used for phylogenetic analysis. (DOCX) [file pone.0064646.s003.docx]

Table S2. Character state scoring for presence-absence (+/-) of clinical amounts of muscarine and psilocybin, and species and GenBank accession numbers used for phylogenetic analysis.

| **Species** | **Muscarine**  **(+/-)** | **Psilocybin**  **(+/-)** | **25S ribosomal RNA GenBank accession no.** | **Voucher no.** |
| --- | --- | --- | --- | --- |
| *Auritella aureoplumosa* | ? | - | AY635766 | Wat23132 |
| *A. brunnescens* nom. prov. | - | - | JQ313571 | PBM3174 |
| *A. dolichocystis* | ? | - | AY380370 | Trappe24844 |
| *A. foveata* | ? | - | GU062739 | TBGT9631 |
| *A. geoaustralis* | ? | - | AY380395 | H7344 |
| *A. serpentinocystis* | - | - | AY038325 | Trappe25080 |
| *Astrosporina alpigenes* | ? | - | AM882750 | EL4905 |
| *Cortinarius iodes* | - | - | AY702013 | PBM2426 |
| *Crepidotus applanatus* | - | - | AF205694 | MCA170 |
| *Inocybe acaciae* nom. prov. | ? | - | JN974921 | NB00356 |
| *I. actinocephala* nom. prov. | ? | - | JN975012 | PBM2863 |
| *I. actinospora* nom. prov. | ? | - | AY380363 | D25 |
| *I. acutella* | ? | - | AM882923 | EL5505 |
| *I. adaequata* | - | - | AM882706 | MR00022 |
| *I. aeruginascens* | - | + | JN974970 | JG270502 |
| *I. aestiva* nom. prov. | ? | - | EU600847 | BK18-Aug-97-6 |
| *I. agardhii* | + | - | AY380366 | JV13740 |
| *I. agglutinata* | + | - | AY038312 | PBM1352 |
| *I. alabamensis* | ? | - | AY536280 | PBM1892 |
| *I. albodisca* | - | ? | EU307819 | PBM1390 |
| *I. albodisca* aff. 1 | - | - | JN974978 | REH7995 |
| *I. albodisca* aff. 2 | - | - | JN974979 | PBM3336 |
| *I. albovestita* | ? | - | EU555443 | PDD71309 |
| *I. alnea* | ? | - | HQ641095 | PAM07090608 |
| *I. amazoniensis* | ? | - | JN642237 | MCA3142 |
| *I. ambigua* | ? | - | AM882800 | BJ910730 |
| *I. ambigua* | ? | - | HQ641096 | PAM08082208 |
| *I. ambigua* cf. | ? | - | AM882796 | EL8105 |
| *I. angustifolia* 1 | ? | - | EU569851 | DED8043 |
| *I. angustifolia* 2 | ? | - | GQ892943 | DED8146 |
| *I. antillana* aff. 1 | ? | - | EU555441 | BZ4124 (DJL79) |
| *I. antillana* aff. 2 | ? | - | EU555442 | STG366 (Lodge SJ15) |
| *I. apiosmota* 1 | ? | ? | JN975022 | AU10560 |
| *I. apiosmota* 2 | ? | ? | JN975021 | PBM3020 |
| *I. appendiculata* | - | - | JN974946 | SAT-00-261-55 |
| *I. armeniaca* 1 | ? | - | AY380367 | PBM1228 |
| *I. armeniaca* 2 | ? | - | JN974952 | SAT0630802 |
| *I. assimilata* | + | - | AM882929 | EL14805 |
| *I. assimilata* cf. | + | - | AM882930 | EL2304 |
| *I. asterospora* 1 | ? | - | AM882897 | MR0015 |
| *I. asterospora* aff. 1 | ? | - | JN974975 | DJL-TN06-58 |
| *I. asterospora* aff. 2 | ? | - | AY702015 | PBM2453 |
| *I. asterospora* cf. | ? | - | AM882722 | EL14305 |
| *I. ayangannae* | ? | - | AY239018 | MCA1232 |
| *I. bakeri* | ? | - | EU307827 | SAT-02-077-01 |
| *I. bicolorata* nom. prov. | ? | - | GQ892938 | ZT12187 |
| *I. bicornis* nom. prov. | ? | - | EU555447 | E5563 |
| *I. bohemica* nom. prov. | - | + | JN974963 | KB04072008 |
| *I. bongardii* | - | - | AM882941 | EL12304 |
| *I. brevisquamulosa* nom. prov. | ? | - | GQ892974 | ZT10102 |
| *I. breviterincarnata* nom. prov. | ? | - | EU555451 | BK28-Aug-04-7 |
| *I. brunneodisca* nom. prov. | ? | - | JN974959 | PBM3220 |
| *I. bulbosissima* | ? | - | AM882765 | EL6605 |
| *I. caerulata* nom. prov. | - | ? | JQ313560 | PBM3127 |
| *I. calamistrata* 1 | - | - | AM882944 | EL13004 |
| *I. calamistrata* 2 | - | - | AM882938 | EL1904 |
| *I. calamistrata* 3 | - | - | EU555452 | JV11950 |
| *I. calamistrata* 4 | - | - | AY380368 | PBM2351 |
| *I. calamistrata* 5 | - | ? | EU555454 | PBM2784 |
| *I. calamistrata* 6 | ? | ? | JN975018 | REH8420 |
| *I. calamistrata* aff. 1 | ? | ? | JN975019 | REH7961 |
| *I. calamistrata* aff. 2 | - | - | FJ627030 | UBC F16562 |
| *I. calamistrata* cf. | ? | ? | AM882945 | EL7703 |
| *I. calamistrata* var. *mucidiolens* | ? | ? | HQ201340 | DG1824 |
| *I. calamistratoides* | ? | ? | AY380369 | ZT96/30 |
| *I. calida* | ? | - | AM882760 | TAA185175 |
| *I. calopedes* | + | - | JQ313572 | PBM3385 |
| *I. calospora* 1 | ? | - | AM882759 | EL9505 |
| *I. calospora* 2 | ? | - | AY038313 | JFA12539 |
| *I. candidipes* | ? | - | AY239019 | BK24-July-99-7 |
| *I. castanea* | ? | - | JN974930 | PBM2115 |
| *I. catalaunica* | ? | - | EU499607 | BK15-Nov-98-7 |
| *I. cerasphora* | ? | - | AY380370 | BSI01/184 |
| *I. cerocarpae* nom. prov. | ? | - | EU600890 | BK20-June-98-6 |
| *I. cervicolor* 1 | - | - | AF347101 | EL27-99 |
| *I. cervicolor* 2 | - | - | AM882939 | SJ04024 |
| *I. chalcodoxantha* | ? | - | HQ201342 | DG1817 |
| *I. chelanensis* | ? | - | AY239021 | PBM2314 |
| *I. chondroderma* nom. prov. | + | - | JN974967 | PBM1776 |
| *I. cincinnata* | + | - | AM882850 | EL6205 |
| *I. cincinnata* aff. | + | - | JN974935 | PBM2641 |
| *I. cinnamomea* | + | - | HQ201345 | PBM564 |
| *I. citrifolia* nom. prov. | ? | - | JN974933 | MGW596 |
| *I. conspicuospora* | ? | - | EU555471 | PC96042 |
| *I. cookei* 1 | ? | - | AM882952 | EL10404 |
| *I. cookei* 2 | ? | - | AM882956 | EL10904 |
| *I. corydalina* | - | + | AY038314 | TURA6488 |
| *I. crucifera* | ? | - | JN974994 | PAM08090318 |
| *I. curreyi* sensu Hesler | ? | - | HQ201348 | PBM2871 |
| *I. curvipes* | + | - | AY239022 | PBM2401 |
| *I. cyanotincta* nom. prov. | ? | ? | JN975033 | I37 |
| *I. diabolica* | ? | - | HQ201350 | JV5712F |
| *I. diminuta* cf. | ? | - | JN974973 | PBM3287 |
| *I. dulcamara* 1 | + | - | AY038315 | BK3-June-99-2 |
| *I. dulcamara* 2 | + | - | AY700196 | JV19652F |
| *I. dulcamara* 3 | + | - | EU569834 | JV7987 |
| *I. dulcamara* 4 | + | - | EU569836 | T10763 |
| *I. emergens* | ? | - | EU569838 | E6203 |
| *I. enigmatica* | ? | - | EU600897 | MCA1490 |
| *I. epidendron* | ? | - | EU569840 | MCA1880 |
| *I. erinaceomorpha* | ? | + | AM882735 | EL12805 |
| *I. errata nom*. prov. | ? | - | EU569844 | DED8022 |
| *I. erubescens* | + | - | AM882951 | KGN980714 |
| *I. exilis* cf. | ? | - | AM882914 | EL3805 |
| *I. fallax* | ? | - | JN974981 | JCS071005D |
| *I. fastigiata* | ? | - | DQ071697 | FO 46800 |
| *I. fastigiella* 1 | ? | - | AY380374 | JRH408 |
| *I. fastigiella* 2 | ? | - | EU569847 | PBM2569 |
| *I. fibrillosibrunnea* | ? | - | EU307848 | E5971 |
| *I. fibrillosibrunnea* aff. | + | - | JN974918 | NB00273 |
| *I. fibrosa* | ? | - | AM882846 | EL2599 |
| *I. fibrosoides* | - | - | AM882827 | SS2990 |
| *I. fibrosoides* cf. | ? | - | JN975001 | PBM2987 |
| *I. flavella* 1 | ? | - | AM882774 | BJ920829 |
| *I. flavella* 2 | ? | - | AM882782 | EL11805 |
| *I. flavella* 3 | ? | - | AM882776 | EL13705 |
| *I. flocculosa* | + | - | AY380375 | PBM2392 |
| *I. flocculosa* aff. | + | - | JN974941 | MB120308 |
| *I. fraudans* | - | - | AM882731 | EL11604 |
| *I. fraudans* aff. | - | - | EU433887 | JFA11831 |
| *I. fuligineoatra* | ? | - | EU307831 | PBM2662 |
| *I. fulvilubrica* | ? | - | EU569850 | PBM2134 (E6776) |
| *I. fuscescentipes* nom. prov. | ? | - | JN974996 | PC96097 |
| *I. fuscidula* 1 | + | - | AM882894 | EL14405 |
| *I. fuscidula* 2 | + | - | AM882886 | EL9505 |
| *I. fuscidula* cf. | + | - | AM882842 | EL1604 |
| *I. fuscodisca* | ? | - | AY380376 | PBM1950 |
| *I. geophylla* 1 | + | - | JN974951 | CA1882 |
| *I. geophylla* 2 | + | - | AM882877 | EL8003 |
| *I. geophylla* 3 | + | - | AY380377 | JV6374 |
| *I. geophylla* cf. | + | - | JN974953 | REH7879 |
| *I. giacomi* | ? | - | AM882742 | EL3105 |
| *I. glabripes* | ? | - | AM882971 | EL8103 |
| *I. glabrodisca* | ? | - | AY239023 | PBM2109 |
| *I. glaucodisca* | ? | - | EU569853 | PC96081 |
| *I. godeyi* | - | - | AY038316 | JV14914F |
| *I. grammata* | - | - | JN974977 | PBM2602 |
| *I. granulosipes* | - | - | JN974917 | E8260 |
| *I. graveolens* | ? | - | JN974983 | PBM3080 |
| *I. griseolilacina* 1 | + | - | AM882728 | EL6704 |
| *I. griseolilacina* aff. 1 | + | - | AY380378 | PBM2241 |
| *I. griseolilacina* aff. 2 | + | - | EU307828 | PBM2661 |
| *I. griseovelata* | ? | - | JN974938 | PBM2442 |
| *I. griseovelata* cf. | ? | - | AM882975 | TAA185125 |
| *I. gymnocarpa* | ? | - | AM882866 | SJ980707 |
| *I. haemacta* | - | + | AM882737 | SJ88062 |
| *I. hebes* nom. prov. | ? | - | JN974997 | PC96110 |
| *I. heimii* | ? | - | AY380379 | JV14932F |
| *I. hirsuta* var. *maxima* | - | ? | EU569854 | PBM2222 |
| *I. hirtella* 1 | + | - | AM882932 | EL12505 |
| *I. hirtella* 2 | + | - | AM882934 | MR00025 |
| *I. hirtella* cf. | + | - | EU307826 | PBM2594 |
| *I. horakomyces* | ? | - | EU600854 | PDD71140 |
| *I. hydrocybiformis* | ? | - | GQ892971 | ZT10077 |
| *I. hydrocybiformis* aff. | ? | - | GQ892973 | DED8165 |
| *I. hystrix* | ? | - | AM882810 | SJ020824 |
| *I. hystrix* aff. | ? | - | JN974969 | REH7405 |
| *I. impexa* | ? | - | AM882821 | TAA172127 |
| *I. incarnata* | - | - | AM882730 | EL15105 |
| *I. infirma* nom. prov. | ? | - | GQ892941 | ZT10106 |
| *I. inodora* | ? | - | AM882834 | EL2405 |
| *I. intricata* | ? | - | EU307835 | PBM2600 |
| *I. ionocephala* nom. prov. (“*lilacina*”) | + | - | JN974950 | MTS2488 |
| *I. jacobi* | ? | - | JN974976 | PBM652 |
| *I. jurana* sensu Hesler | + | - | HQ201353 | PBM2951 |
| *I. kauffmanii* | + | - | JN974948 | PBM1796 |
| *I. lacera* 1 | + | - | AM882823 | EL2104 |
| *I. lacera* 2 | + | - | AY038318 | PBM1462 |
| *I. lacera* 3 | + | - | JN974993 | PBM2541 |
| *I. lacera* var. *helobia* | + | - | AM882815 | EL15605 |
| *I. laetior* | ? | - | HQ201355 | Stz4655 |
| *I. lanatodisca* 1 | - | - | JN975034 | PBM2588 |
| *I. lanatodisca* 2 | ? | - | AY380382 | SAT-99-229-01 |
| *I. langei* | + | - | JN974962 | JV13640F |
| *I. lanuginosa* | + | - | AY038319 | PBM956 |
| *I. lasseri* | ? | - | EU569857 | MCA1971 |
| *I. latericia* | ? | ? | JN975024 | ZT8481 |
| *I. latericia* aff. | ? | ? | JN975023 | TR109-02 |
| *I. latibulosa* nom. prov. | ? | - | GQ892947 | DED8054b |
| *I. leiocephala 1* | ? | - | AY380383 | JV9448 |
| *I. leiocephala* 2 | + | - | GQ906703 | PBM1569 |
| *I. lepidotella* | ? | - | JN642234 | MCA1881 |
| *I. leptocystis* | ? | - | AM882801 | SJ96002 |
| *I. leptophylla* 1 | ? | - | AM882787 | BJ920801 |
| *I. leptophylla* 2 | ? | - | AY038320 | BK7-Sept-97-19 |
| *I. leucoblema* | ? | - | EU569858 | JV16437 |
| *I. lilacina* 1 | + | - | AM882875 | EL12605 |
| *I. lilacina* 2 | + | - | AM882873 | EL9205 |
| *I. lilacina* 3 | + | - | AF042616 | JM96/25 |
| *I. lilacina* 4 | + | - | AY380385 | PBM2039 |
| *I. lilacina* 5 | + | - | JN974949 | PBM2716 |
| *I. lilacinosquamosa* | ? | - | AY380386 | MCA1464 |
| *I. lineata* nom. prov. | ? | - | GQ892958 | DED8048 |
| *I. luteifolia* | - | - | EU307814 | PBM2642 |
| *I. luteobulbosa* | ? | - | JQ313573 | PDD75276 |
| *I. maculata* 1 | + | - | AM882964 | EL12604 |
| *I. maculata* 2 | ? | - | AY745700 | PBM2446 |
| *I. maculata* 3 | ? | - | EU569868 | PBM2542 |
| *I. maculata* 4 | ? | - | JN975026 | PBM3051 |
| *I. maculata* 5 | ? | - | JN975025 | SAT-04-274-06 |
| *I. magnifolia* | ? | - | EU600900 | MCA2441 |
| *I. malenconii* | + | - | EU569870 | JV5498A |
| *I. margaritispora* aff. | ? | - | JN974998 | MR00198 |
| *I. marginata* | + | - | AY509115 | MCA1882 |
| *I. melanopus* | + | - | AM882725 | BJ920904 |
| *I. microlepidota* nom. prov. | ? | - | JN974961 | PBM578 |
| *I. microspora* cf. | ? | - | AM882808 | TAA185187 |
| *I. mimica* | ? | - | AM882781 | TK2004114 |
| *I. misakaensis* | - | - | EU569874 | PC96234 |
| *I. mixtilis* 1 | + | - | AM882836 | EL8904 |
| *I. mixtilis* 2 | + | - | HQ641115 | PAM07110104 |
| *I. mixtilis* 3 | + | - | AY380387 | PBM1315 |
| *I. mixtilis* aff. | + | - | JN975000 | PBM2455 |
| *I. monticola* | ? | - | EU499608 | BK28-June-98-3 |
| *I. mucidiolens* | ? | ? | AY038317 | PBM1066 |
| *I. muricellata* | ? | - | AM882915 | EL3704 |
| *I. murrayana* cf. | ? | - | EU555461 | PBM2180 (E7029) |
| *I. mutata* | ? | ? | AY732212 | PBM2542 |
| *I. mytiliodora* | - | - | JN974947 | PBM1572 |
| *I. napipes* | + | - | AY239024 | PBM2376 |
| *I. neglecta* nom. prov. | ? | - | EU600829 | DED8063 |
| *I. nematoloma* | ? | - | AM882713 | EL10105 |
| *I. nitidiuscula* | + | - | AM882847 | EL11804 |
| *I. niveivelata* nom. prov. aff. | ? | - | JN975007 | MCA704 |
| *I. nothopedes* nom. prov. | ? | - | AY380388 | Trappe25060 |
| *I. oblectabilis* | + | - | AM882831 | BJ920908 |
| *I. oblectabilis* cf. | ? | - | HQ641099 | PAM00110404 |
| *I. obscurobadia* | ? | - | AM882802 | SJ03008 |
| *I. obsoleta* | ? | - | AM882769 | EL1704 |
| *I. obtusiuscula* | ? | - | HQ641112 | PAM02081710 |
| *I. occidentalis* nom. prov. (“cf. *rimosa*”) | ? | - | AY038321 | PBM525 |
| *I. ochroalba* | ? | - | AM882882 | EL5704 |
| *I. olympiana* | + | - | EU307841 | PBM2219 |
| *I. ornata* nom. prov. | ? | - | GQ892975 | ZT10107 |
| *I. pallidobrunnea* aff. | ? | - | JN974968 | PBM2242 |
| *I. palliflavipes* nom. prov. | ? | - | JQ313574 | PBM3434 |
| *I. parvibulbosa* 2 | ? | - | GQ892955 | ZT10099 |
| *I. parvibulbosa* 3 | ? | - | GQ892957 | ZT10105 |
| *I. parvibulbosa* 4 | ? | - | GQ892954 | DED8021 |
| *I. parvibulbosa* nom. prov. 1 | ? | - | GQ892956 | ZT10078 |
| *I. pelargonium* | + | - | AM882936 | TK04063 |
| *I. perlata* | - | - | AM882771 | EL7404 |
| *I. perlata* aff. | ? | - | JN975013 | PBM2982 |
| *I. persicinipes nom. prov.* | ? | - | EU600837 | PBM2197 (E7044) |
| *I. petiginosa* | ? | - | AM882708 | EL6304 |
| *I. phaeodisca* cf. | ? | - | AM882978 | SJ95012 |
| *I. phaeoleuca* 1 | ? | - | EU499617 | BK27-Aug-97-1 |
| *I. phaeoleuca* 2 | ? | - | EU499618 | TURA171481 |
| *I. phaeosquarrosa* | ? | - | JQ313575 | PBM3087 |
| *I. phaeosticta* 1 | ? | - | HQ641102 | PAM05091310 |
| *I. phaeosticta* 2 | ? | - | HQ641100 | PAM95082312 |
| *I. pileosulcata* aff. | ? | - | GQ892952 | DED8163 |
| *I. pileosulcata* nom. prov. | + | - | EU600838 | DED8058 |
| *I. posterula* | ? | - | AM882868 | EBJ051120 |
| *I. praecox* | ? | - | AY038311 | PBM1402 |
| *I. praetervisa* | + | - | AM882720 | EL12904 |
| *I. praetervisa* cf. | + | - | AM882721 | SJ95027 |
| *I. proxima* nom. prov. | ? | - | EU600840 | DED8007 |
| *I. pseudoasterospora* | ? | - | AM882921 | SJ880430 |
| *I. pseudorachodes* aff. | ? | - | JN974936 | BAP565 |
| *I. pseudoreducta* aff. | ? | - | AM882970 | EL9804 |
| *I. pudica* | + | - | AY038323 | PBM1373 |
| *I. pulchella* | ? | - | JN642241 | MCA2270 |
| *I. pusillima* 1 | ? | - | GQ892959 | DED8145 |
| *I. pusillima* 2 | ? | - | GQ892960 | ZT10130 |
| *I. pusio* 1 | + | - | AY388643 | PBM2297 |
| *I. pusio* 2 | + | - | JN974955 | PBM2627 |
| *I. pyriodora* var. *chamaesalicis* | ? | - | AM882733 | EL6405 |
| *I. queletii* 1 | + | - | EU307813 | JV19682F |
| *I. queletii* 2 | + | - | AY380390 | PBM935 |
| *I. quietiodor* | ? | - | AM882960 | EL11504 |
| *I. redolens* nom. prov. | ? | - | EU600844 | PBM2185 (E7034) |
| *I. regisii* nom. prov. | ? | - | JN975027 | PC96082 |
| *I. reisneri* cf. | ? | - | EU555463 | MCA646 |
| *I. relicina* | ? | - | AY038324 | JV10258 |
| *I. renispora* aff. 1 | ? | - | EU555466 | PBM2195 (E7042) |
| *I. renispora* aff. 2 | ? | - | AY732213 | PBM2206 (E7054) |
| *I. rennyi* | ? | - | AM882715 | SJ88034 |
| *I. rimosa* 1 | + | - | AM882762 | EL7505 |
| *I. rimosa* 2 | ? | - | EF561633 | PBM2574 |
| *I. rimosa* aff. | ? | - | EU600853 | PBM2654 |
| *I. rimosa* aff. | ? | - | JN975005 | TR183-05 |
| *I. rimosa* cf. | ? | - | AM882786 | EL7104 |
| *I. rimosa* cf. | ? | - | JN975006 | PBM2958 |
| *I. rimosoides* | - | - | AY702014 | PBM2459 |
| *I. saliceticola* | ? | - | AM882717 | BJ9008270 |
| *I. salicis* | ? | - | AM882724 | EL7105 |
| *I. salicis-herbaceae* | ? | - | AY038322 | PBM1021 |
| *I. sambucina* | ? | - | AM882757 | SJ01002 |
| *I. scissa* | + | - | JQ313576 | PBM3387 |
| *I. scissa* cf. | ? | - | JN974985 | PDD75859 |
| *I. semifulva* | ? | - | HQ222007 | ACAD11651 |
| *I. serotina* | + | - | AY380391 | NI210995 |
| *I. serrata* | ? | - | EU600856 | E6320 |
| *I. sierraensis* | ? | - | AY239025 | DED6101 |
| *I. sindonia* | + | - | AY380393 | PBM2048 |
| *I. sindonia* aff. | + | - | EU499601 | BK01-Nov-98-8 |
| *I. sinuospora* nom. prov. | ? | - | EU600861 | E5980 |
| *I. soluta* 1 | + | - | AM882755 | EL2904 |
| *I. soluta* 2 | + | - | JN974987 | JV7811F |
| *I. sororia* | + | - | EU600863 | JRH661 |
| *I. sororia* aff. | ? | - | JN975004 | REH8245 |
| *I. spadicea* nom. prov. | ? | - | EU600865 | PBM2203 (E7051) |
| *I. sphaerospora* | ? | - | GQ892948 | DED8059 |
| *I. sphaerospora* aff. | ? | - | GQ892949 | DED8153 |
| *I. splendens* 1 | ? | - | GQ892828 | AF3130 |
| *I. splendens* 2 | ? | - | EU499603 | BK09-Nov-98-8 |
| *I. splendens* 3 | ? | - | EU499611 | TURA147679 |
| *I. splendens* 4 | ? | - | EU499613 | TURA171482 |
| *I. spuria* | + | - | AM882784 | SJ92017 |
| *I. squarrosa* | ? | - | AM882790 | SJ93026 |
| *I. stellata* 1 | ? | - | GQ892967 | ECV3648 |
| *I. stellata* 2 | ? | - | GQ892963 | ZT10097 |
| *I. stellata* 3 | ? | - | GQ892962 | ECV3651 |
| *I. stellata* aff. | ? | - | GQ892964 | ZT10123 |
| *I. stellatospora* | ? | - | EU307840 | PRL2716 |
| *I. strigiceps* | ? | - | EU555469 | PDD71260 |
| *I. subcarpta* | + | - | AM882754 | EL8905 |
| *I. subexilis* 1 | ? | - | AM882711 | BJ910730 |
| *I. subexilis* 2 | - | - | EU307845 | PBM2620 |
| *I. subfulva* | ? | - | JN974989 | PBM1482 |
| *I. subochracea* 1 | - | - | AY380397 | PBM1143 |
| *I. subochracea* 2 | - | - | JN974971 | PBM2673 |
| *I. subochracea* 3 | - | - | JN974972 | SH083007 |
| *I. subporospora* | ? | - | AM882931 | RP950618 |
| *I. tabacina* | ? | - | HQ641106 | PAM05071302 |
| *I. tahquamenonensis* | - | - | AY380399 | PBM1142 |
| *I. tenebrosa* 1 | ? | - | AM882967 | EL8204 |
| *I. tenebrosa* 2 | ? | - | JN974945 | JV14910 |
| *I. teraturgus* 1 | ? | - | AY239027 | JV4290 |
| *I. teraturgus* 2 | ? | - | AM882789 | SJ79017 |
| *I. terrigena* | - | - | AM882864 | EL11704 |
| *I. tetragonospora* | ? | - | AM882748 | EL1505 |
| *I. thailandica* nom. prov. | ? | - | GQ892968 | DED8049 |
| *I. torresiae* nom. prov. | ? | - | EU600874 | PBM2157 (E6978) |
| *I. tricolor* | - | + | AM882738 | SJ05011 |
| *I. tubarioides* | ? | - | AY732211 | PBM2550 |
| *I. umbratica* 1 | + | - | AM882798 | SJ03020 |
| *I. umbratica* 2 | ? | - | AY732209 | PBM2552 |
| *I. umbrinella* | ? | - | JN975010 | PBM3024 |
| *I. umbrosa* aff. | ? | - | EU555468 | PBM2184 (E7033) |
| *I. unicolor* 1 | - | - | AY380403 | PBM1481 |
| *I. unicolor* 2 | - | - | AF518622 | RV7/4 |
| *I. vestipides* nom. prov. | ? | - | EU600859 | PBM2199 (E7046) |
| *I. violaceocaulis* | ? | - | AY732208 | PBM2614 (E7013) |
| *I. viscata* | - | - | JQ313570 | PBM3445 |
| *I. vulpiniceps* nom. prov. | ? | - | EU600879 | PBM1922 |
| *I. xanthomelas* aff. | - | - | HQ641104 | PAM07062202 |
| *I. xerophytica* | + | - | EU600880 | GUA-242 |
| *Inocybe* sp. 1 | ? | - | JN974944 | a200101801-28 |
| *Inocybe* sp. 2 | ? | - | EU600877 | ADP960305 |
| *Inocybe* sp. 3 | ? | - | JN975030 | ALW5108b |
| *Inocybe* sp. 4 | ? | - | EU600885 | BB3233 (PC) |
| *Inocybe* sp. 5 | ? | - | EU600831 | BK21-Aug-97-18 |
| *Inocybe* sp. 6 | ? | - | AY038326 | BK8-Feb-99-1 |
| *Inocybe* sp. 7 | ? | - | JN974988 | BZ480 |
| *Inocybe* sp. 8 | ? | - | AF261510 | DAOM174733 “*I. petiginosa*” |
| *Inocybe* sp. 9 | ? | - | GQ892950 | DED8044 |
| *Inocybe* sp. 10 | ? | - | GQ892953 | DED8054a |
| *Inocybe* sp. 11 | ? | - | GQ892977 | DED8061 |
| *Inocybe* sp. 12 | ? | - | GQ892970 | DED8065 |
| *Inocybe* sp. 13 | ? | - | GQ892978 | DED8133 |
| *Inocybe* sp. 14 | ? | - | GQ892937 | DED8134 |
| *Inocybe* sp. 15 | ? | - | GQ892939 | DED8161 |
| *Inocybe* sp. 16 | ? | - | EU600851 | DJL-SJ14 |
| *Inocybe* sp. 17 | ? | - | EU600894 | E7066 |
| *Inocybe* sp. 18 | ? | - | JN975029 | EC300 |
| *Inocybe* sp. 19 | ? | - | EU600895 | GDa |
| *Inocybe* sp. 20 | ? | - | JN974922 | GDsn |
| *Inocybe* sp. 21 | ? | - | JN975016 | MCA562 |
| *Inocybe* sp. 22 | ? | - | JN974982 | MCA705 |
| *Inocybe* sp. 23 | ? | - | JN975008 | MTS2494 |
| *Inocybe* sp. 24 | ? | - | JN975002 | NB00186 |
| *Inocybe* sp. 25 | ? | - | JN974925 | NB00256 |
| *Inocybe* sp. 26 | ? | - | JN974995 | NB00258 |
| *Inocybe* sp. 27 | ? | - | JN974919 | NB00352 |
| *Inocybe* sp. 28 | ? | - | EU307834 | NI250904 |
| *Inocybe* sp. 29 | ? | - | JN974942 | PBM1921 |
| *Inocybe* sp. 30 | ? | - | EU555456 | PBM2125 (E6758) |
| *Inocybe* sp. 31 | ? | - | EU569843 | PBM2132 (E6768) |
| *Inocybe* sp. 32 | ? | - | EU307851 | PBM2181 (E7030) |
| *Inocybe* sp. 33 | ? | - | EU600834 | PBM2350 |
| *Inocybe* sp. 34 | ? | - | JN974940 | PBM2352 |
| *Inocybe* sp. 35 | ? | - | EU307818 | PBM2449 |
| *Inocybe* sp. 36 | ? | - | EU600852 | PBM2601 |
| *Inocybe* sp. 37 | ? | - | JN974954 | PBM2633 |
| *Inocybe* sp. 38 | ? | - | JN974929 | PBM2783 |
| *Inocybe* sp. 39 | ? | - | JN974934 | PBM2909 |
| *Inocybe* sp. 40 | ? | - | JN975031 | PBM2963 |
| *Inocybe* sp. 41 | ? | - | JN975015 | PBM2983 |
| *Inocybe* sp. 42 | ? | - | JN974999 | PBM3050 |
| *Inocybe* sp. 43 | ? | - | JN974924 | PBM3079 |
| *Inocybe* sp. 44 | ? | - | JN974923 | PBM3086 |
| *Inocybe* sp. 45 | ? | - | JN974927 | PBM3110 |
| *Inocybe* sp. 46 | ? | - | JN974926 | PBM3115 |
| *Inocybe* sp. 47 | ? | - | JN974974 | PBM3123 |
| *Inocybe* sp. 48 | ? | - | JN974920 | PBM3131 |
| *Inocybe* sp. 49 | ? | - | JN974966 | PBM3334 |
| *Inocybe* sp. 50 | ? | - | JN974990 | PBM3335 |
| *Inocybe* sp. 51 | ? | - | EU600883 | PC96013 |
| *Inocybe* sp. 52 | ? | - | EU555474 | PC96039 |
| *Inocybe* sp. 53 | ? | - | EU600870 | PC96073 |
| *Inocybe* sp. 54 | ? | - | EU600884 | PC96083 |
| *Inocybe* sp. 55 | ? | - | EU569860 | PC96095 |
| *Inocybe* sp. 56 | ? | - | EU600875 | PC96111 |
| *Inocybe* sp. 57 | ? | - | EU569871 | PC96204 |
| *Inocybe* sp. 58 | ? | - | JN974956 | PDD77716 |
| *Inocybe* sp. 59 | ? | - | JN974939 | REH7020 |
| *Inocybe* sp. 60 | ? | - | JN974980 | REH7181 |
| *Inocybe* sp. 61 | ? | - | JN974931 | REH7418 |
| *Inocybe* sp. 62 | ? | - | JN974932 | REH7965 |
| *Inocybe* sp. 63 | ? | - | JN974991 | REH8914 |
| *Inocybe* sp. 64 | ? | - | EU307837 | SA100602A |
| *Inocybe* sp. 65 | ? | - | EU307838 | SA100602B |
| *Inocybe* sp. 66 | ? | - | JN974986 | SAT-06-294-06 |
| *Inocybe* sp. 67 | ? | - | JN974937 | SAT-07-125-06 |
| *Inocybe* sp. 68 | ? | - | EU555457 | T18260 |
| *Inocybe* sp. 69 | ? | - | EU522732 | TM02_127 |
| *Inocybe* sp. 70 | ? | - | EU522733 | TM02_130 |
| *Inocybe* sp. 71 | ? | - | EU522754 | TM02_239 |
| *Inocybe* sp. 72 | ? | - | EU522755 | TM02_24 |
| *Inocybe* sp. 73 | ? | - | EU522765 | TM02_288 |
| *Inocybe* sp. 74 | ? | - | EU522766 | TM02_3 |
| *Inocybe* sp. 75 | ? | - | EU522782 | TM02_37 |
| *Inocybe* sp. 76 | ? | - | EU522787 | TM02_66 |
| *Inocybe* sp. 77 | ? | - | EU522796 | TM02_93 |
| *Inocybe* sp. 78 | ? | - | EU522845 | TM03_497 |
| *Inocybe* sp. 79 | ? | - | JN975011 | TR104-05 |
| *Inocybe* sp. 80 | ? | - | JN975009 | TR138-05 |
| *Inocybe* sp. 81 | ? | - | JN974964 | TR170-02 |
| *Inocybe* sp. 82 | ? | - | JN974958 | TR176-05 |
| *Inocybe* sp. 83 | ? | - | JN974965 | TR180-02 |
| *Inocybe* sp. 84 | ? | - | JN975032 | TR194-02 |
| *Inocybe* sp. 85 | ? | - | JN975017 | TR220-06 |
| *Inocybe* sp. 86 | ? | - | JN974928 | TR4-07 |
| *Inocybe* sp. 87 | ? | - | JN974960 | TR44-06 |
| *Inocybe* sp. 88 | ? | - | JN974943 | TR46-06 |
| *Inocybe* sp. 89 | ? | - | JN975014 | TR49-05 |
| *Inocybe* sp. 90 | ? | - | JN975003 | TR70-04 |
| *Inocybe* sp. 91 | ? | - | JN974957 | TR71-05 |
| *Inocybe* sp. 92 | ? | - | JN975020 | TR74-06 |
| *Inocybe* sp. 93 | ? | - | JN974992 | TR88-06 |
| *Inocybe* sp. 94 | ? | - | AY380389 | Trappe24874 |
| *Inocybe* sp. 95 | ? | - | JN974984 | Trappe31970 |
| *Inocybe* sp. 96 | ? | - | GQ892976 | ZT10031 |
| *Inocybe* sp. 97 | ? | - | EU600903 | ZT8944 |
| *Inocybe* sp. 98 | ? | - | EU604546 | ZT9250 |
| *Mallocybe armillata* nom. prov. | ? | - | EU555446 | PBM2290 |
| *M. flavicothurnata* nom. prov. | ? | - | AY038327 | PBM1615 |
| *M. jarrahae* nom. prov. | ? | - | AY380381 | PBM2207 |
| *M. subtilisior* cf. | ? | - | JN974916 | E8178 |
| *M. subtilisior* nom. prov. | ? | - | AY380398 | OKM24631 |
| *Mallocybe* sp. 1 | ? | - | AY380400 | BK-6-Jun-97-24 |
| *Mallocybe* sp. 2 | ? | - | AY380394 | PBM2397 |
| *Mallocybe* sp. 3 | ? | - | JN975028 | Sayers s.n. |
| *Tubariomyces hygrophoroides* | ? | - | GU907094 | P05112008 |
| *T. inexpectatus* | - | - | EU569855 | AH20390 |
| *Tubariomyces* sp. 1 | ? | - | EU600887 | BB6018 |
| *Tubariomyces* sp. 2 | ? | - | GU907092 | RFS0805 |
| Uncultured basidiomycete | ? | ? | FJ566015 | A11YG20Rm1 |
| Uncultured basidiomycete | ? | ? | FJ566087 | A11YK01RM1 |
| Uncultured basidiomycete | ? | ? | FJ566668 | A1YE16RM1 |
| Uncultured basidiomycete | ? | ? | FJ566934 | A2YA06RM1 |
| Uncultured basidiomycete | ? | ? | FJ567000 | A2YD07RM1 |
| Uncultured basidiomycete | ? | ? | FJ567053 | A2YF15RM1 |
| Uncultured basidiomycete | ? | ? | FJ567088 | A2YH02RM1 |
| Uncultured basidiomycete | ? | ? | FJ567187 | A2YL11RM1 |
| Uncultured basidiomycete | ? | ? | FJ567207 | A2YM07RM1 |
| Uncultured basidiomycete | ? | ? | FJ567238 | A2YN18RM1 |
| Uncultured basidiomycete | ? | ? | FJ567351 | A7YC21RM1 |
| Uncultured basidiomycete | ? | ? | FJ567525 | A7YK20RM1 |
| Uncultured basidiomycete | ? | ? | FJ567609 | A7YO20RM1 |
| Uncultured basidiomycete | ? | ? | FJ568031 | A9YB17RM1 |
| Uncultured basidiomycete | ? | ? | FJ568209 | A9YK02RM1 |
| Uncultured basidiomycete | ? | ? | FJ568283 | A9YK06RM1 |
| Uncultured basidiomycete | ? | ? | FJ568319 | A9YP03RM1 |
| Uncultured Cortinariaceae | ? | ? | DQ273511 | K4 |
| Uncultured ectomycorrhiza | ? | ? | EF417792 | B1BA3 |
| Uncultured ectomycorrhiza | ? | ? | DQ974812 | Bg8d |
| Uncultured ectomycorrhiza | ? | ? | EF417809 | L3BB12 |
| Uncultured ectomycorrhiza | ? | ? | EF417810 | L3BD11 |
| Uncultured ectomycorrhiza | ? | ? | FM993253 | L3X29 |
| Uncultured ectomycorrhiza | ? | ? | EF417812 | L4BA2 |
| Uncultured ectomycorrhiza | ? | ? | FN557549 | L6133_Inoc_Y01 |
| Uncultured ectomycorrhiza | ? | ? | FJ196928 | M42B1 |
| Uncultured ectomycorrhiza | ? | ? | EU563510 | M43A4 |
| Uncultured ectomycorrhiza | ? | ? | AY748869 | MUN9 |
| Uncultured fungus | ? | ? | EU522868 | 4c |
| Uncultured fungus | ? | ? | EU522899 | Ss1 |
| Uncultured *Inocybe* | ? | ? | FJ207485 | 121_2 |
| Uncultured *Inocybe* | ? | ? | FJ207477 | 13_1 |
| Uncultured *Inocybe* | ? | ? | FJ207510 | B17_1 |
| Uncultured soil fungus | ? | ? | EU861785 | FunCON4_12C |
| Uncultured soil fungus | ? | ? | EU691943 | MWCt11T8_7A |
| Uncultured soil fungus | ? | ? | EU692212 | MWGM1T8_1E |
